# Host‐Specific Interactions Between *Staphylococcus aureus* and *Pseudomonas aeruginosa* Impair Epithelial Repair in Chronic Rhinosinusitis

**DOI:** 10.1002/alr.70228

**Published:** 2026-08-03

**Authors:** Xiaohan Sun, Mahnaz Ramezanpour, George Bouras, Emma Barry, Alkis J. Psaltis, Peter‐John Wormald, Sarah Vreugde

**Affiliations:** ^1^ Department of Surgery‐Otolaryngology Head and Neck Surgery Central Adelaide Local Health Network Woodville South Australia Australia; ^2^ Adelaide Medical School Faculty of Health and Medical Sciences The University of Adelaide Adelaide South Australia Australia

**Keywords:** chronic rhinosinusitis, epithelial repair, host‐specific bacterial interaction, oxidative stress, polymicrobial infections, *Pseudomonas aeruginosa*, *Staphylococcus aureus*

## Abstract

**Background:**

Chronic rhinosinusitis (CRS) is frequently associated with polymicrobial biofilms involving *Staphylococcus aureus* and *Pseudomonas aeruginosa*. Interactions between these organisms are thought to influence disease severity, but the epithelial effects of exoproteins derived from patient‐matched cocultures remain poorly defined.

**Methods:**

Clinical isolates of *S. aureus* and *P. aeruginosa* (*n* = 3 each) co‐isolated from three CRS patients were cultured in a Transwell system as same‐patient or cross‐patient pairs. Cell‐free exoproteins were applied to primary human nasal epithelial cells. Epithelial repair was assessed using a scratch assay, while cytotoxicity, oxidative stress, and inflammatory responses were evaluated by lactate dehydrogenase release, intracellular reactive oxygen species measurement, and interleukin‐6 secretion. Exoprotein profiles were further characterized using data‐independent acquisition proteomics.

**Results:**

Exoproteins derived from same‐patient cocultures consistently impaired epithelial wound closure compared with *P. aeruginosa* monocultures, although the timing of inhibition varied among patients. These exoproteins also induced greater epithelial cytotoxicity, elevated intracellular reactive oxygen species levels, and increased interleukin‐6 secretion compared with monocultures or cross‐patient cocultures. In contrast, cross‐patient pairings produced epithelial responses similar to monoculture conditions. Proteomic analysis indicated that patient origin was a major determinant of exoproteome organization, with same‐patient cocultures showing increased abundance of proteins associated with redox balance and metabolic regulation.

**Conclusions:**

Patient‐matched *S. aureus*–*P. aeruginosa* interactions were associated with increased epithelial stress and inflammatory responses, together with differences in the exoproteomic profile. These findings suggest that host‐specific bacterial interactions may contribute to virulence and possibly recalcitrance in CRS.

## 1 Introduction

Chronic rhinosinusitis (CRS) is characterized by chronic inflammation of the sinonasal mucosa linked to a loss of mucosal barrier function, complex microbial biofilm communities, significant quality‐of‐life implications, and major health system burdens worldwide [1]. *Staphylococcus aureus* and *Pseudomonas aeruginosa* can coinfect in more severe forms of treatment‐resistant CRS, suggestive of interactive pathogenic effects with implications for disease pathogenesis and treatment [2, 3].

Previous research has emphasized the role of microbial interspecies relationships in polymicrobial biofilm communities, demonstrating a marked influence on virulence factor expression, biofilm stability, antibiotic susceptibility, and the associated inflammatory responses in hosts [4, 5]. In particular, the influence of *S. aureus* and *P. aeruginosa* coinfection on microbial ecosystem dynamics has been studied in the context of cystic fibrosis [6, 7, 8]. These two bacterial species have been found to have both competitive and synergistic relationships, leading potentially to altered microbial ecosystem dynamics, virulence expression, and antibiotic resistance profiles [9]. However, given that such opposing relationships have been shown, further research is required to understand whether and how their interactions might influence the pathophysiology of CRS [10].

Bacterial exoproteins, such as proteases, toxins, and immune modulators, play pivotal roles in mediating the complex interplay between the host and microbes, thereby affecting the integrity of the epithelial barrier, immune system activation, and mucosal repair mechanisms [11]. Human nasal epithelial cells (HNECs), acting as physical barriers and the body's first line of immune defense, exhibit high capability in secreting cytokines in addition to mediating mucosal repair mechanisms following bacterial stimulations [12]. Interleukin‐6 (IL‐6), a key pro‐inflammatory cytokine, has been largely implicated in mediating the inflammatory cascade in CRS, thus providing an indispensable marker for assessing inflammatory responses within the sinonasal compartment [1, 13]. Additionally, the repair mechanisms within the sinonasal epithelial cells, largely measured using an in vitro scratch wound‐healing assay, present an indispensable marker of the mucosa's repair capabilities in CRS [14].

Despite the well‐recognized importance of bacterial interactions in chronic inflammatory diseases, comprehensive functional studies evaluating the direct impact of exoproteins from cocultured *S. aureus* and *P. aeruginosa*, specifically isolated from CRS patients, remain rare [15]. Very few studies have explored how these coculture‐derived exoproteins affect epithelial wound healing, which is a key component of mucosal barrier function and is frequently impaired in CRS. The nature of species‐to‐species communication within patient‐derived polymicrobial biofilms may therefore play an important role in shaping epithelial repair and host inflammatory responses, ultimately contributing to disease persistence [16]. In addition, the current literature provides limited proteomic insight into these bacterial relationships, making it difficult to identify strain‐specific factors that account for the observed phenotypic variability [17, 18]. For these reasons, it is necessary to investigate both the functional effects on wound healing and the proteomic characteristics of these interactions to better understand bacterial adaptability in chronic inflammatory diseases such as CRS.

The objective of this study is to explore the functional roles of bacterial exoproteins derived from cocultured *S. aureus* and *P. aeruginosa* isolates harvested simultaneously from CRS sufferers using efficient epithelial cell models. By doing so, we aimed to determine the identity and effect of bacterial secreted factors on the kinetics of wound closure, as well as their toxicity and inflammatory responses. Knowledge of the host‐specific microbial interactions and proteomics correlates is essential for understanding the pathogenesis of CRS, in addition to providing insights into the development of personalized therapies.

## 2 Methods and Materials

### 2.1 Bacterial Strains

The bacterial strains and primary HNECs used in this study were ethically obtained with approval from the Human Research Ethics Committee of the Central Adelaide Local Health Network (reference: HREC/18/CALHN/69). All subjects provided written informed consent prior to participation. CRS patients were enrolled based on diagnostic criteria specified by the latest guidelines from the American Academy of Otolaryngology–Head and Neck Surgery as well as the European Position Paper on Rhinosinusitis and Nasal Polyps (EPOS) [19]. Identification of bacterial isolates, specifically *S. aureus* and *P. aeruginosa*, was performed using matrix‐assisted laser desorption ionization–time of flight mass spectrometry (MALDI–TOF MS) (Bruker MBT, SA Pathology, Adelaide, Australia). Post‐identification, these strains were stored at −80°C in a mixture of tryptone soy broth (TSB) (Oxoid, Basingstoke, UK) and 20% sterile glycerol (v/v) until needed.

Patient‐specific clinical data, including CRS phenotype, comorbidities, and disease severity (Adelaide Disease Severity Score [ADSS]), were obtained as described previously [15].

### 2.2 Isolation and Cultivation of Primary HNECs

Primary HNECs were obtained via minimally invasive endoscopic brushing from the middle turbinate of patients clinically diagnosed with CRS. Harvested cells were initially suspended in nasal epithelial–specific growth medium (STEMCELL Technologies Australia Pty. Ltd., Tullamarine, Victoria, Australia). To eliminate monocyte contamination, the cell suspension was incubated on culture dishes pre‐coated with anti‐CD68 antibodies (Dako, Glostrup, Denmark). Subsequent expansion of HNECs was performed in collagen Type I‐coated culture flasks (Thermo Scientific, Waltham, Massachusetts, USA) using PneumaCult‐EX Plus Complete Medium (Stemcell Technologies, 05001). Culturing conditions included incubation at 37°C in a humidified atmosphere containing 5% CO_2_. Cells at their first passage were used for subsequent experimental protocols.

### 2.3 Preparation of Exoproteins From Cocultures

Overnight cultures of clinical isolates of *S. aureus* (C317, C318, C324) and *P. aeruginosa* (C440, C442, C443) previously described in our earlier study [15] grown on tryptic soy agar (TSA) plates were suspended in 0.9% NaCl and adjusted to a 1.0 ± 0.1 McFarland standard (≈3 × 10^8^ CFU/mL), then diluted 1:15 in nutrient broth. For all coculture experiments, both bacterial species were grown in the same TSB medium. For coculture, 100 µL of *S. aureus* (from the same or a different patient) was added to the apical chamber and 500 µL of *P. aeruginosa* was added to the basal chamber of Transwell 24‐well plates (Corning, New York, USA). Plates were incubated at 37°C for 48 h on a 3D Gyratory Mixer (Ratek Instruments, Boronia, Victoria, Australia). Basal supernatants were then collected, centrifuged at 1500 × *g* for 10 min at 4°C, and filtered through 0.22‐µm Acrodisc syringe filters (Life Science, Fribourg, Switzerland). The filtrates were concentrated using 3‐kDa Pierce Protein Concentrators (Thermo Fisher Scientific, Rockford, Illinois, USA). Protein concentrations were quantified using the Nano‐Orange Protein Quantitation Kit (Molecular Probes, Eugene, Oregon, USA), and fluorescence was measured with excitation at 485 nm and emission at 590 nm using a FLUOstar Optima microplate reader (BMG Labtech, Ortenberg, Germany).

### 2.4 Scratch Assay With Coculture Exoprotein Treatment

HNECs were seeded into IncuCyte‐compatible culture plates (Sartorius, Göttingen, Germany), pretreated with collagen Type I (Corning, cat. no. 354249, Bedford, Massachusetts, USA) and cultured for 48 h in PneumaCult‐EX Plus complete medium (Stemcell Technologies, 05001) until reaching 90%–95% confluence, as confirmed by phase‐contrast microscopy. Standardized wounds were created using the IncuCyte WoundMaker (Sartorius), followed by gentle media replacement to remove debris. Exoproteins were assessed across 1–5 µg/mL to define a biologically relevant working concentration. Preliminary dose–response experiments identified 3 µg/mL as the optimal concentration, as it produced reproducible epithelial injury without complete loss of viability, thereby enabling detection of strain‐specific differences [20]. For all subsequent experiments, primary HNECs were treated with 3‐µg/mL exoproteins derived from *P. aeruginosa* monocultures or from cocultures of *P. aeruginosa* with either patient‐matched or non‐matched *S. aureus* isolates. Exoproteins from *P. aeruginosa* monocultures served as the reference control for comparison of coculture effects, while cells treated with PneumaCult‐EX Plus complete medium alone served as the negative control. Plates were then transferred to the IncuCyte live‐cell imaging system, and wound closure was monitored over 24 h under standard conditions (37°C, 5% CO_2_). Wound healing dynamics were quantified using IncuCyte analysis software to assess differences across treatment groups. Cell‐free supernatants were collected and used for quantification of lactate dehydrogenase (LDH) release, reactive oxygen species (ROS) production, and IL‐6 secretion by enzyme‐linked immunosorbent assay [19].

### 2.5 LDH Cytotoxicity Assay

The assay was performed using the CytoTox 96 Non‐Radioactive Cytotoxicity Assay Kit (Promega, Madison, Wisconsin, USA) according to the manufacturer's protocol. Briefly, 50 µL of each sample were transferred into duplicate wells of a flat‐bottom 96‐well plate (Corning, New York, USA). Then 50 µL of reconstituted substrate mixture was added into each well and left to incubate away from light at room temperature. After 30 min of incubation, 50 µL of stop solution was added to each well and the absorbance was measured at 490 nm using a FLUOstar Optima microplate reader (BMG Labtech, Ortenberg, Germany).

### 2.6 ROS Detection Assay

Intracellular ROS levels were assessed in parallel using the same scratch injury model described above. Following cell seeding and treatment with 3‐µg/mL exoproteins under identical experimental conditions, cells were incubated with 10 µM Carboxy‐H2DCFDA (10 µM; Invitrogen, cat. no. D399, Carlsbad, California, USA) in PBS for 60 min at 37°C in the dark. After washing with PBS, fluorescence was measured directly in the same plate at 492/525 nm using a FLUOstar Optima plate reader (BMG Labtech, Ortenberg, Germany). Detector gain was set automatically to maintain values within the linear range. ROS levels were expressed as fold change relative to the PneumaCult‐Ex Plus medium (Ex‐Plus) control to account for baseline fluorescence variation.

### 2.7 IL‐6 Quantification by ELISA

IL‐6 protein levels were quantified using a commercially available sandwich ELISA kit (BD Biosciences, New Jersey, USA) according to the manufacturer's instructions, as previously described [21]. Samples were analyzed in duplicate, and absorbance was measured using a FLUOstar Optima plate reader (BMG Labtech, Ortenberg, Germany).

#### 2.7.1 Exoprotein Preparation and Liquid Chromatography Mass Spectrometry (LC–MS/MS) Acquisition

Exoproteins were obtained from *P. aeruginosa* monocultures and *S. aureus*–*P. aeruginosa* cocultures, including homotypic (SAx/PAx) and heterotypic (SAy/PAx) isolate pairs. Cell‐free supernatants were concentrated using 10‐kDa centrifugal filters (Amicon Ultra, Millipore), washed in PBS, and stored at −80°C. Protein concentrations were determined using the NanoOrange Protein Quantitation Kit (Thermo Fisher Scientific, Waltham, Massachusetts, USA).

Protein processing was performed according to our previously established quantitative proteomic workflow [22]. Briefly, proteins were reduced (TCEP, 10 mM, 56°C, 30 min), alkylated (chloroacetamide, 20 mM, 30 min; Sigma–Aldrich, St. Louis, Missouri, USA), and cleaned using an SP3‐based magnetic bead protocol (Sera‐Mag, Cytiva, 24152105050250, Marlborough, Massachusetts, USA). Samples were digested overnight with trypsin (1:20; Promega, Madison, Wisconsin, USA), reprecipitated in ≥ 95% acetonitrile, washed, eluted in 2% DMSO, dried, and resuspended in 0.1% formic acid to 1 µg/3 µL. Equal aliquots were pooled for chromatogram library generation.

### 2.8 LC–MS/MS and DIA Analysis

Peptides were analyzed on a Vanquish Neo UHPLC coupled to an Orbitrap Eclipse 480 mass spectrometer (Thermo Fisher Scientific). Separation was performed on a 25‐cm in‐house packed C18 column (75 µm ID, 3 µm ReproSil‐Pur 120 C18‐AQ resin; Dr. Maisch) using a 120‐min gradient (3%–40% acetonitrile in 0.1% formic acid). Spectral libraries were generated from pooled digests using DDA with gas‐phase fractionation (350–1200 m/z; MS1 60,000; MS2 15,000; HCD NCE 30%; dynamic exclusion 10 s). DIA was acquired using 50 variable isolation windows spanning 350–1200 m/z (MS1 60,000; MS2 30,000; HCD NCE 30%), following our previously established workflow [22]. Given the well‐recognized heterogeneity in sampling and mass spectrometry methodologies across CRS proteomic studies, we used a single standardized acquisition and processing pipeline for all samples to maximize comparability [23].

DIA data were processed in Spectronaut using default settings with a *q*‐value threshold of 0.05 for peptide and protein identification, based on a combined protein database comprising *P. aeruginosa* (in‐house curated pangenome database) and *S. aureus* (UniProtKB, release 2024_02). Downstream analyses were performed using complementary workflows in Python and R as indicated in Section 2.9 below [22].

### 2.9 Statistical Analysis

Functional assay data (scratch assay, LDH release, ROS production, and IL‐6 ELISA) were analyzed using GraphPad Prism (v10). Comparisons between groups were performed using one‐ or two‐way ANOVA with appropriate post hoc correction. Data are presented as mean ± SD, with *p* < 0.05 considered statistically significant.

Proteomic data were analyzed in Python (pandas, numpy, scikit‐learn, matplotlib, seaborn, and scikit‐bio) using a protein intensity matrix that had been preprocessed with MinProb imputation. Protein intensities were log2‐transformed, and the top 25% most variable proteins were retained for principal component analysis (PCA). Data were mean‐centered prior to PCA, which was performed using the scikit‐learn implementation based on singular value decomposition (SVD). The first two principal components were used to visualize sample clustering and variance structure, with group distributions illustrated by covariance‐based confidence ellipses. Group differences were assessed using PERMANOVA with dispersion testing.

Differential protein abundance was determined using moderated linear modeling (limma) in R [24]. Given the exploratory nature of the analysis and the limited sample size, nominal *p* < 0.05 together with a |log2 fold change| ≥ 0.5 threshold was applied to identify differentially expressed proteins.

## 3 Results

The study included three paired *P. aeruginosa* and *S. aureus* clinical isolates co‐isolated from the same nasal swab of three CRS patients. These patients displayed distinct CRS phenotypes and comorbidities, with varying degrees of disease severity as summarized in Table 1.

**TABLE 1:** Overview of clinical and demographic features of CRS patients providing co‐isolated *P. aeruginosa* and *S. aureus* strains.

Patient ID	Age (years)	Sex	CRS phenotype	Smoking history	Allergies	Asthma	GORD	DM	ADSS
6027	82	F	CRSsNP	Nonsmoker	None	Yes	Yes	No	25
539	60	F	CRSwNP	Nonsmoker	Yes	Yes	Yes	Yes	14.5
1415	53	M	CRSwNP	Unknown	None	No	Yes	Yes	Not recorded

Abbreviations: ADSS, Adelaide Disease Severity Score; CRS, chronic rhinosinusitis; CRSsNP, CRS without nasal polyps; CRSwNP, CRS with nasal polyps; DM, diabetes mellitus; F, female; GORD, gastroesophageal reflux disease; M, male.

### 3.1 Same‐Patient *S. aureus* and *P. aeruginosa* Cocultures Impaired the Wound Healing of HNECs

We first sought to determine whether exoproteins derived from *S. aureus* and *P. aeruginosa* cocultures influence the ability of epithelial cells to actively repair damage. To address this, we employed a scratch assay to monitor wound closure dynamics in primary HNEC monolayers.

In our pilot study, exoproteins derived from same‐patient cocultures consistently impaired epithelial wound closure across all tested concentrations (1–5 µg/mL), without exhibiting a consistent dose‐dependent trend (Figure S1). Based on this reproducibility and to ensure comparability across experiments, 3 µg/mL was selected as a representative concentration for all further analyses.

Exoproteins from all paired isolates significantly reduced wound closure compared with media control, with the earliest significant effect observed at 4 h (*p* < 0.01) and impairment persisting to 24 h (*p* < 0.05). Two‐way ANOVA with Tukey's post hoc analysis across the three isolate sets was then conducted to evaluate whether same‐patient isolates differentially affected wound closure compared to monocultures and to heterologous cocultures. For all three patients, exoproteins derived from *P. aeruginosa* cocultured with *S. aureus* isolated from the same patient significantly reduced the wound healing compared to *P. aeruginosa* monocultures and to heterologous cocultures at 24 h (*p* < 0.05). The onset of differentiation varied with differences between matched isolates and monocultures seen as early as 6 h (Patient 1) and as late as the 24‐h time point (Patient 3). For all three patients, cocultures with heterologous *S. aureus* strains produced significantly higher closure percentages compared with the *P. aeruginosa* monocultures for at least one non‐matched *S. aureus* at 24 h (*p* < 0.05) (Figure 1 and Table S1).

**FIGURE 1:**
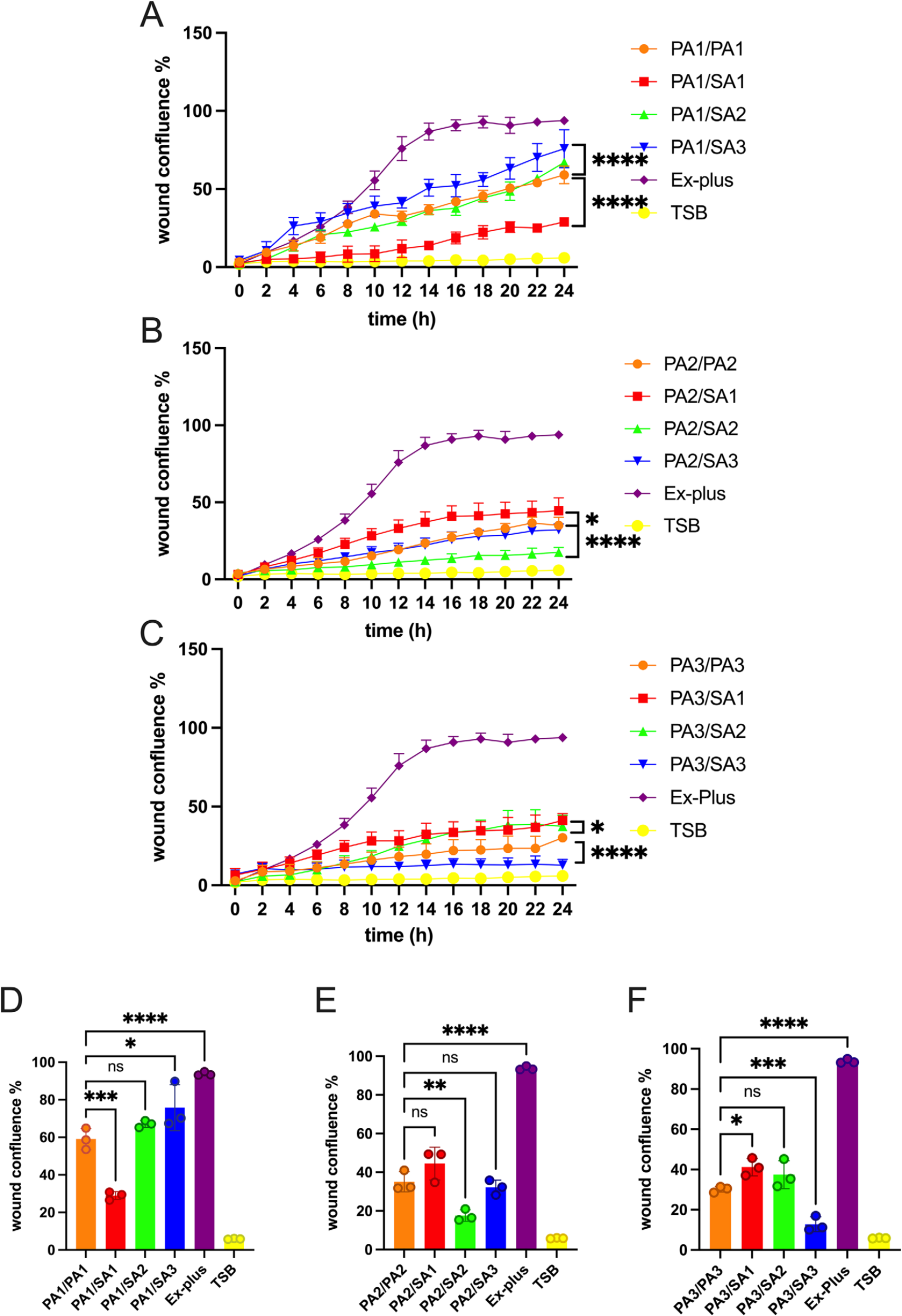
Wound closure dynamics in scratch assays with exoproteins from monocultures and cocultures. (A–C) Time‐course analysis of wound closure in primary HNEC monolayers treated with exoproteins (3 µg/mL) derived from *P. aeruginosa* monocultures and *P. aeruginosa/ S. aureus* cocultures with *S. aureus* from same or different patients for Patient (A) 1, (B) 2, and (C) 3. Wound closure was monitored over 24 h using live‐cell imaging. (D–F) Endpoint analysis of wound confluence percentage at 24 h in monocultures of *P. aeruginosa* and cocultures of *S. aureus* from the same or different patients and *P. aeruginosa* for Patient (D) 1, (E) 2, and (F) 3. *PA–SA*: *P. aeruginosa* is cultured in the lower chamber while *S. aureus* is cultured in the upper chamber. Statistical significance was determined using one‐way ANOVA with Dunnett's post hoc test and two‐way ANOVA with Tukey's post hoc test. ns, not significant;*PA*, *P. aeruginosa; SA*, *S. aureus*. *p < 0.05; **p < 0.01; ***p < 0.001; ****p < 0.0001.

### 3.2 Matched Coculture Exoproteins Increase Cytotoxicity, Oxidative Stress, and Inflammatory Signaling

We next wanted to evaluate whether impaired wound healing was linked to cytotoxic effects, ROS production, and inflammatory responses. Across all three patient isolate sets, exoproteins derived from matched cocultures consistently reduced epithelial viability, increased ROS production, and increased IL‐6 production compared with Ex‐Plus medium–treated control cells (*p* < 0.05). Moreover, these effects were consistently more pronounced than those observed with *P. aeruginosa* monocultures or heterologous cocultures (*p* < 0.05) (Figure 2 and Tables S2–S4).

**FIGURE 2:**
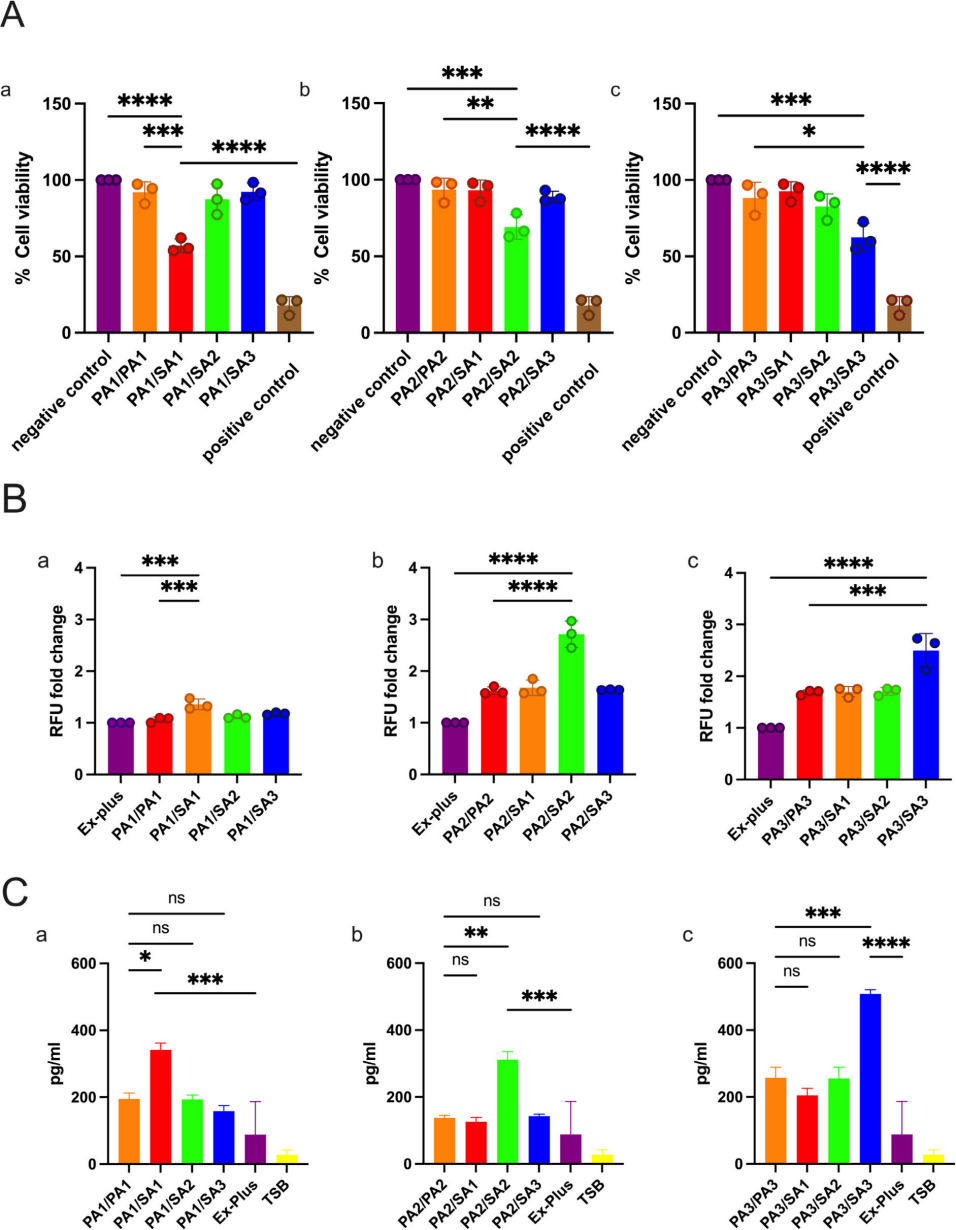
Assessment of epithelial cytotoxicity, oxidative stress, and inflammatory response following exposure to bacterial exoproteins. (A) Cell viability calculated from LDH release in primary human nasal epithelial cells (HNECs) 24 h after treatment with bacterial exoproteins (3 µg/mL), expressed as a percentage relative to the negative control (Ex‐Plus), which was set to 100%. (B) Intracellular ROS levels measured 24 h after exoprotein exposure and expressed as fold change in relative fluorescence units (RFU) normalized to the PneumaCult‐EX Plus control. (C) IL‐6 secretion quantified in cell supernatants after 24 h by sandwich ELISA and expressed as pg/mL. For each panel, subpanels (a–c) correspond to isolates from (a) Patient 1, (b) Patient 2, and (c) Patient 3. *PA–SA*: *P. aeruginosa* is cultured in the lower chamber while *S. aureus* is cultured in the upper chamber. For the LDH assay, the negative control was HNECs treated with Ex‐Plus medium alone, and the positive control was the LDH high‐control reagent provided in the assay kit. Data represent *n* = 3 independent biological replicates and are presented as mean ± SD. Statistical significance was determined by one‐way ANOVA with Tukey's multiple comparisons test. ns, not significant; *PA, P. aeruginosa; SA*, *S. aureus*. *p < 0.05; **p < 0.01; ***p < 0.001; ****p < 0.0001.

### 3.3 Global Organization of the Exoproteome Landscape

We then analyzed the proteomes of 12 exoprotein fractions derived from *P. aeruginosa* monocultures and *P. aeruginosa*–*S. aureus* cocultures, including both same‐patient and cross‐patient isolate pairs, using DIA LC–MS/MS.

PCA of all exoprotein datasets showed substantial overlap among experimental groups (Figure 3A,B) (full PCA input matrix is provided as Data S1 in. tsv format). When samples were colored by functional category (same patient, cross patient, and *PA* mono), only limited separation was observed in PCA space (Figure 3A). The first two principal components explained 61.0% of the total variance (PC1 = 49.9%, PC2 = 11.1%). Cross patient samples were distributed along a diagonal axis across PC1 and PC2, whereas same patient samples occupied a broader region primarily along PC1. *PA* mono samples were more tightly clustered at lower PC2 values, although partial overlap with other groups remained. PERMANOVA based on Euclidean distances showed no significant group separation (Pseudo‐*F* = 0.95, *p*_perm = 0.431), and PERMDISP indicated no difference in dispersion among groups (*F* = 1.28, *p* = 0.218). Substantial overlap between functional categories was observed in PCA space.

**FIGURE 3:**
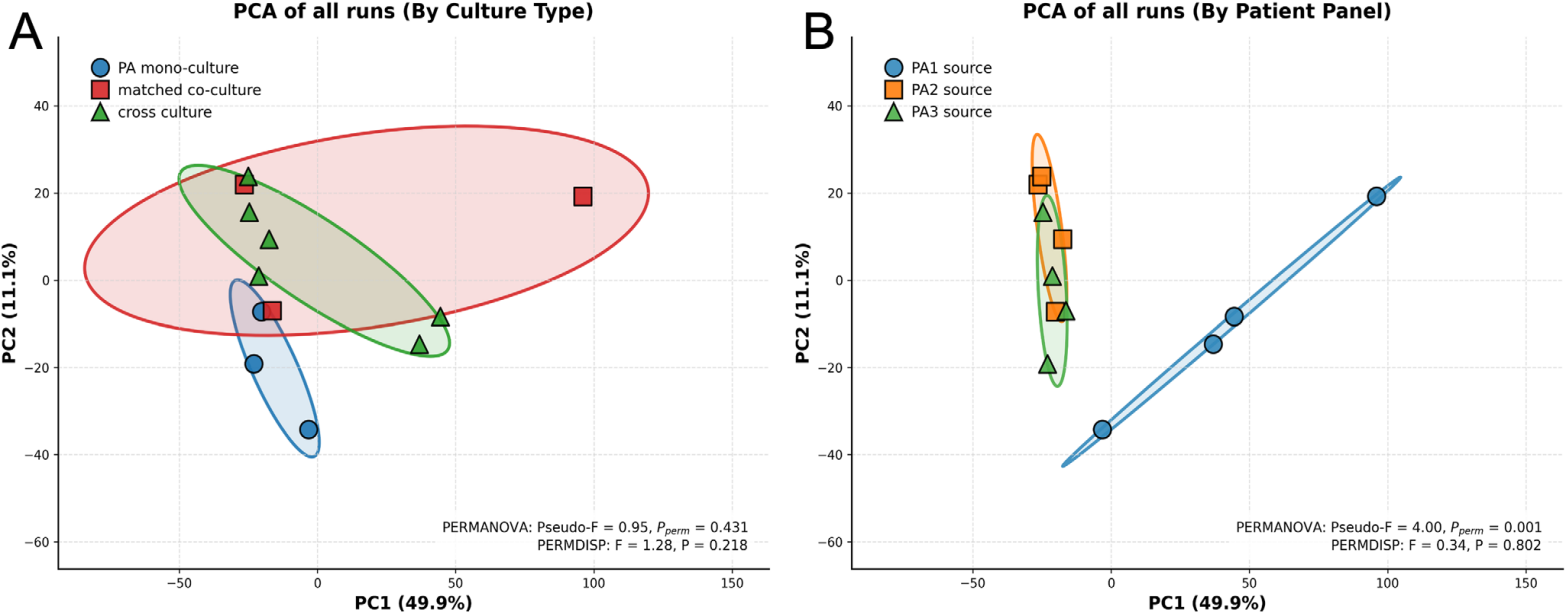
PCA and functional enrichment of exoprotein profiles. (A–B) PCA plots of all exoprotein samples. Each point represents one exoproteome, and ellipses indicate group distributions. (A) Samples grouped as matched coculture, cross‐culture, and *PA* monoculture. (B) Samples grouped by patient origin (PA1, PA2, PA3).

When samples were regrouped by patient origin (PA1–PA3) (Figure 3B), a distinct distribution pattern was observed. PA1 samples formed a linear distribution along PC1, whereas PA2 and PA3 samples were more compactly clustered. PERMANOVA indicated differences between patient groups (Pseudo‐*F* = 4.00, *p*_perm = 0.001), while PERMDISP showed no difference in dispersion (*F* = 0.34, *p* = 0.802). Variation associated with patient origin was reflected in the PCA distribution.

Differential protein abundance analysis between matched *S. aureus*–*P. aeruginosa* cocultures and *P. aeruginosa* monocultures identified a distinct set of altered proteins (Figure 4A and Data S2). Proteins increased in the matched coculture included Group_1685 (kinase family protein), NarG (nitrate reductase alpha subunit), Dat (*D*‐amino acid transaminase), Group_419 (reductase), PdhD (dihydrolipoamide dehydrogenase), FabZ (3‐hydroxyacyl‐[acyl‐carrier‐protein] dehydratase), SodM (superoxide dismutase [Mn]), MntC (manganese transport system substrate‐binding protein), DeoD (purine nucleoside phosphorylase), Gap (glyceraldehyde‐3‐phosphate dehydrogenase), Pgm (phosphoglycerate mutase), and HemC (porphobilinogen deaminase), whereas CvfB (RNA‐binding regulatory protein CvfB) was decreased. The upregulated proteins included multiple enzymes associated with redox‐related processes, central metabolic pathways, and protein modification.

**FIGURE 4:**
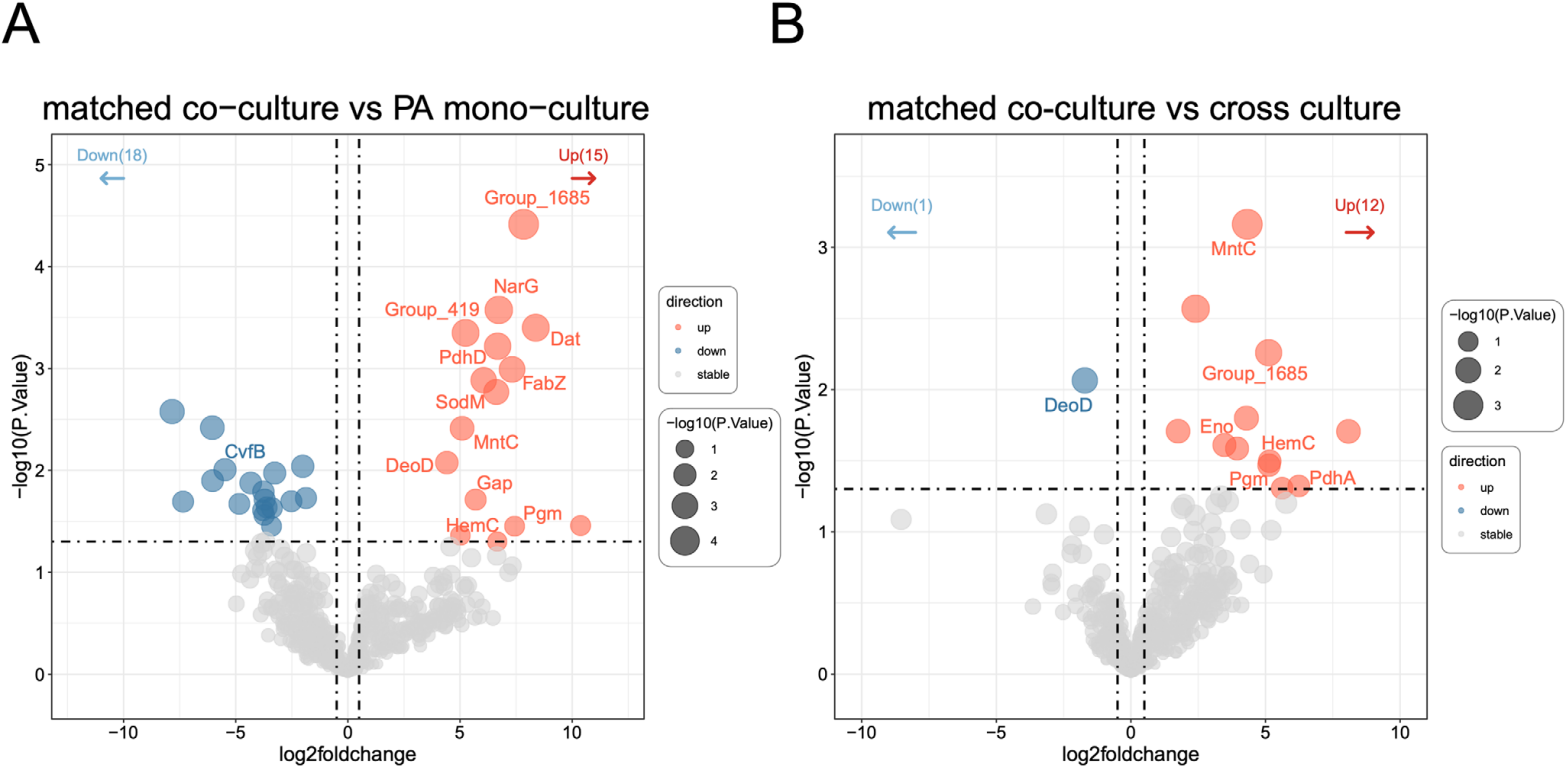
Differential protein expression of exoprotein profiles. (A–B) Volcano plots of differentially abundant proteins between groups. Point size indicates −log10 (nominal *p* value) and point color indicates log2 fold change. Labeled proteins denote representative significant changes. (A) Matched *S. aureus*–*P. aeruginosa* cocultures versus *P. aeruginosa* monocultures. (B) Matched cocultures versus cross‐culture combinations.

When the matched coculture was compared with cross‐culture combinations (Figure 4B and Data S3), a more restricted set of proteins was identified. Among the differentially abundant proteins, several representative proteins remained upregulated, including MntC, Group_1685, Eno, Pgm, HemC, and PdhA (pyruvate dehydrogenase E1 component subunit alpha), whereas DeoD was decreased. Additional differentially abundant proteins were detected but are not individually annotated (Data S3).

## 4 Discussion

This study examined the effects of exoproteins from *S. aureus*–*P. aeruginosa* cocultures on epithelial wound repair, cytotoxicity, oxidative stress, and IL‐6 secretion. Exoproteins from homologous pairings produced greater inhibition of wound closure and higher LDH release, ROS production, and IL‐6 levels than monocultures or cross‐patient combinations. DIA proteomics identified coordinated alterations in the secreted protein profile under homologous conditions, supporting a role for within‐host adaptation in enhancing interspecies virulence.

Under standardized exposure (3 µg/mL, 24 h), homologous exoproteins induced sustained impairment of epithelial repair, indicating enrichment of factors that potentially disrupt migratory and proliferative capacity beyond the effect of *P. aeruginosa* alone [25]. Delayed wound closure reflects functional compromise of epithelial restitution, with reduced capacity for coordinated migration at the wound edge. In scratch assays, this phenotype extends beyond reduced cell movement and implicates disruption of membrane integrity, cytoskeletal organization, cell–cell coordination, and metabolic support required for migration across the denuded surface [26, 27].

Homologous exoproteins were associated with increased LDH release, ROS production, and IL‐6 secretion compared with monocultures and heterologous pairings, defining a state of epithelial stress characterized by concurrent perturbation of membrane integrity, redox balance, and inflammatory activation. Despite assessment at a single 24‐h time point only, the concordant elevation of these parameters aligns with progressive epithelial destabilization and delayed repair observed in the scratch assay. LDH release reflects membrane injury without implying complete epithelial loss, and its moderate elevation alongside impaired wound closure is consistent with a sublethal injury state in which epithelial cells persist but exhibit reduced reparative capacity [28, 29]. Elevated intracellular ROS reflects sustained redox stress during the repair phase [30]. Controlled redox signaling sustains epithelial migration, whereas persistent oxidative pressure disrupts cytoskeletal remodeling, focal adhesion turnover, and collective cell movement [31]. Spatially regulated hydrogen peroxide signaling coordinates actin cytoskeletal dynamics during epithelial repair, placing redox control as a key determinant of restitution. Increased ROS under homologous conditions aligns with impaired cell polarity, lamellipodia extension, and coordinated migration at the wound edge [27]. Increased IL‐6 further reflects epithelial activation coupled to membrane injury and redox imbalance, with inflammatory signaling modulating repair behavior and sustaining mucosal stress [32]. In CRS, epithelial barrier dysfunction, inflammatory heterogeneity, and disrupted repair programs coexist [33], and the combined elevation of LDH, ROS, and IL‐6 delineates an epithelial state characterized by impaired restitution, persistent activation, and reduced capacity for mucosal recovery.

Proteomic profiling of homologous coculture relative to monoculture indicated coordinated metabolic and redox adaptations aligned with this epithelial stress phenotype. A subset of proteins was consistently altered, defining a metabolic–redox axis associated with impaired restitution. Increased SodM [Mn] together with enrichment of the manganese transporter (MntC) is consistent with sustained manganese‐dependent redox regulation, a system linked to oxidative stress handling and cytoskeletal organization [34, 35], and concordant with the elevated ROS and impaired wound closure observed. Additional enrichment of 3‐hydroxyacyl‐ACP dehydratase (FabZ) suggests lipid remodeling processes that may influence membrane composition and epithelial interface interactions [36], aligning with the membrane injury reflected by LDH release. Changes in central metabolic enzymes, including glyceraldehyde‐3‐phosphate dehydrogenase (Gap) and phosphoglycerate mutase (Pgm), together with increases in nitrate reductase (NarG) and porphobilinogen deaminase (HemC), are consistent with shifts in glycolytic flux and respiratory activity linked to redox balance, potentially supporting sustained metabolic activity and redox turnover under coculture conditions [37, 38, 39, 40].

Alongside these metabolic adaptations, regulatory features indicate modulation beyond a uniform increase in virulence‐associated activity. CvfB was reduced in homologous relative to monoculture conditions, consistent with altered RNA‐mediated regulatory pathways [41, 42], while DeoD displayed context‐dependent variation consistent with redistribution of purine metabolism [43, 44]. Group_1685, annotated as a kinase family protein, was also increased, suggesting potential involvement of phosphorylation‐dependent regulatory processes [45].

Comparison between homologous and heterologous cocultures revealed a more restricted proteomic shift, with MntC remaining elevated while SodM did not, indicating persistence of a core redox adaptation without uniform amplification. Enolase may further contribute at the host interface through interactions with plasminogen and extracellular matrix components, potentially influencing matrix remodeling and epithelial activation [46]. These features indicate selective regulatory and host‐interface modulation under homologous conditions.

These observations are consistent with a coordinated metabolic–redox adaptation contributing to a sustained epithelial stress phenotype characterized by membrane injury, oxidative imbalance, and inflammatory activation [47]. At the proteome‐wide level, however, this functional convergence was not accompanied by consistent structural segregation. Indeed, PCA revealed no consistent segregation of global exoproteomic structure by culture condition, with substantial overlap across PA monoculture, matched coculture, and cross‐culture. Patient‐stratified analysis showed variable separation without a consistent directional pattern, indicating absence of a unified organizational structure. This heterogeneity was not mirrored at the functional level. The data are consistent with homologous interactions supporting a metabolic–redox–oriented functional state despite divergent proteomic configurations, resulting in broadly conserved epithelial injury phenotypes across patients.

The present findings support a model in which patient‐matched *S. aureus*–*P. aeruginosa* interactions are associated with distinct exoproteomic responses and enhanced epithelial stress. Although the current study was not designed to establish causality, coordinated alterations in proteins linked to metabolic and redox processes may contribute to the increased LDH release, ROS production, IL‐6 secretion, and impaired wound repair observed under homologous conditions. This proposed relationship is summarized in the schematic illustration (Figure 5). While the mechanisms underlying these patient‐specific effects remain unresolved, the observed exoproteomic differences provide a potential explanation for the greater epithelial stress induced by homologous cocultures compared with cross‐patient cocultures.

**FIGURE 5:**
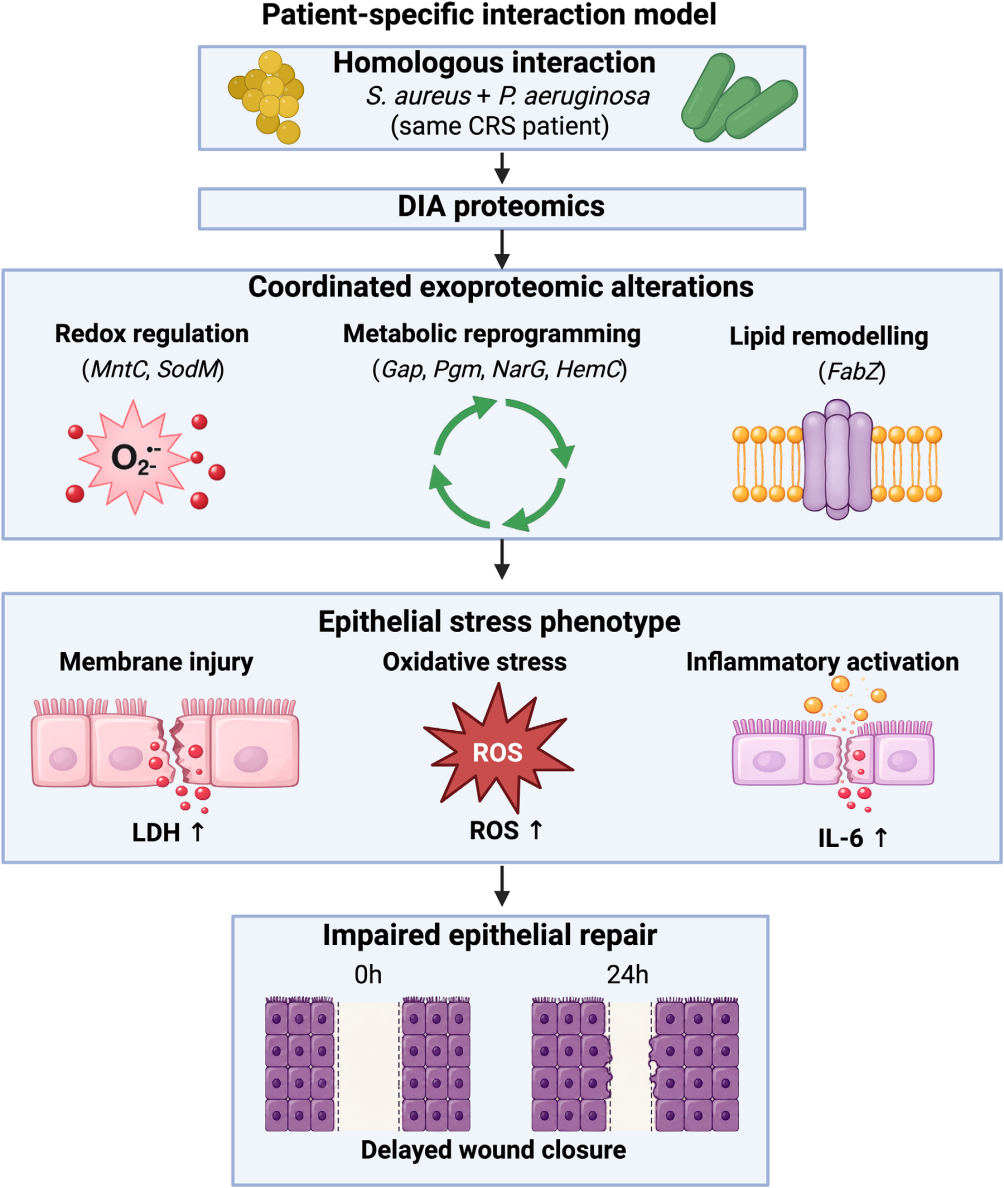
Proposed model of patient‐specific *S. aureus*–*P. aeruginosa* interactions and epithelial stress. Proposed mechanistic relationships remain to be experimentally validated.

Several limitations apply. The number of patient‐derived strain pairs was limited, constraining statistical power and generalizability. Functional assays were performed on composite exoprotein fractions, precluding attribution to individual proteins. The in vitro system, including the use of submerged HNEC monolayers for wound‐healing experiments, does not fully recapitulate the structural and functional characteristics of differentiated ALI cultures or the complexity of the sinonasal environment in vivo. The exoprotein fraction likely includes nonprotein components. Causality between individual factors and epithelial responses was not directly resolved. Further, proteomic analyses relied on nominal *p*‐values within an exploratory framework.

Patient‐matched *S. aureus*–*P. aeruginosa* interactions align with coordinated metabolic–redox processes rather than uniform activation of classical virulence pathways. Alterations in glycolysis and central carbon metabolism support coupling between metabolic flux and redox balance, with potential implications for epithelial homeostasis [48, 49]. Additional signals implicate manganese acquisition, lipid remodeling, and purine metabolism as processes associated with the observed CRS phenotype, suggesting coordinated physiological adaptation during interspecies interaction. These findings provide biological context for the distinct exoproteomic profiles observed under homologous conditions.

## 5 Conclusion

Patient‐matched *S. aureus*–*P. aeruginosa* interactions are associated with enhanced epithelial stress and a consistent injury phenotype, reflecting adaptation within a shared host environment. These findings suggest that host‐shaped bacterial interactions may contribute to persistent epithelial dysfunction in CRS and may represent a potential target for therapeutic intervention.

## Funding

The authors have nothing to report.

## Conflicts of Interest

The authors declare no conflicts of interest.

## Supporting information




**Supporting File 1**: alr70228‐sup‐0001‐FigureS1.tiff

**Supporting File 2**: alr70228‐sup‐0002‐DataS1.tsv

**Supporting File 3**: alr70228‐sup‐0003‐DataS2.csv

**Supporting File 4**: alr70228‐sup‐0004‐DataS3.csv

**Supporting File 5**: alr70228‐sup‐0005‐SupMat.docx
