# Supplementary material for: Host‐Specific Interactions Between Staphylococcus aureus and Pseudomonas aeruginosa Impair Epithelial Repair in Chronic Rhinosinusitis
Source: Int Forum Allergy Rhinol. 2026 Aug 3;16(11):1318–29. doi: 10.1002/alr.70228 (PMC13731154; doi:10.1002/alr.70228)
Supplement: Supplementary file 1 [file ALR-16-1318-s002.docx]

**Supplementary Table S1:** Wound closure differences between *P. aeruginosa* monocultures and *S. aureus*–*P. aeruginosa* co-cultures, expressed as wound confluence percentage.

| Patient | Earliest Significant Time Point | MD ± SE at Earliest Time Point | p-value at Earliest Time Point | MD ± SE at 24h | p-value at 24h | Wound Confluence % at 24h (Mono vs. Co-culture) |
| --- | --- | --- | --- | --- | --- | --- |
| P1 | 6h | 12.59 ± 3.139 | 0.0013 | 30.09 ± 3.139 | <0.0001 | 59.02 ± 3.270 vs. 28.94 ± 1.262 (p=0.0018) |
| P2 | 14h | 11.15 ± 2.924 | 0.0027 | 17.46 ± 2.924 | <0.0001 | 35.07 ± 3.000 vs. 17.62 ± 1.737 (p=0.0020) |
| P3 | 24h | 17.41 ± 3.718 | <0.0001 | 17.41 ± 3.718 | <0.0001 | 30.16 ± 0.827 vs. 12.76 ± 2.114 (p=0.0004) |

**LDH assay demonstrates early cytotoxicity induced by same-patient co-culture exoproteins**

**Supplementary Table S2:** Cell viability (%) measured by LDH assay after 24-hour exposure to exoproteins from *P. aeruginosa* mono-cultures and *S. aureus*–*P. aeruginosa* co-cultures. Data are presented as mean ± SEM. p < 0.05 indicates significant reduction vs. *P. aeruginosa* monoculture, while p > 0.05 indicates no statistically significant difference.

| Patient | Cell Viability (%) - PA+SA Co-culture | Cell Viability (%) - PA Mono | p- value (Co-culture vs. Mono) | p- value (Diff. Pts vs. Mono) |
| --- | --- | --- | --- | --- |
| P1 | 57.04± 2.556 | 92.05 ± 3.873 | 0.0002 | > 0.05 |
| P2 | 69.15 ± 4.634 | 93.57 ± 4.350 | 0.0030 | > 0.05 |
| P3 | 62.41 ± 5.347 | 88.25 ± 5.874 | 0.0104 | > 0.05 |

**Exoproteins from matched *S. aureus*–*P. aeruginosa* pairs induce elevated intracellular ROS levels after 24-hour exposure**

**Supplementary Table S3:** Fluorescence intensity measurements in the basal chamber for paracellular permeability assays after 24-hour incubation with *P. aeruginosa* (PA) mono-cultures or PA co-cultured with *S. aureus* isolates from the same or different patients. Data represent mean fluorescence intensity (RFU) ± standard error. p < 0.05 indicates a significant increase in fluorescence intensity, while p > 0.05 indicates no statistically significant difference.

| Patient | Fluorescence (RFU) - PA+SA Co-culture | Fluorescence (RFU) - PA Mono | p- value (Co-culture vs. Mono) | p- value (Diff. Pts vs. Mono) |
| --- | --- | --- | --- | --- |
| P1 | 572.0 ± 12.00 | 447.3 ± 7.460 | 0.0001 | > 0.05 |
| P2 | 1142 ± 34.14 | 682.4 ± 7.849 | <0.0001 | > 0.05 |
| P3 | 1050 ± 58.52 | 710.4 ± 9.328 | <0.0001 | > 0.05 |

**Exoproteins collected after 24-hour culture from same-patient co-cultures elicit elevated IL-6 secretion in HNECs**

**Supplementary Table S4:** IL-6 secretion in HNECs after 24-hour exposure to bacterial exoproteins. Primary HNECs were treated for 24 hours with 3 µg/ml exoproteins collected from *P. aeruginosa* mono-cultures or co-cultures with *S. aureus* isolates from the same or different patients. IL-6 levels (pg/ml) were quantified by ELISA and are presented as mean ± SEM. p < 0.05 indicates a significant difference compared with *P. aeruginosa* mono-cultures.

| Patient | IL-6 (pg/ml) -  PA+SA Co-culture | IL-6 (pg/ml) -  PA Mono | p- value (Co-culture vs. Mono) | p- value (Diff. Pts vs. Mono) |
| --- | --- | --- | --- | --- |
| P1 | 341.2 ± 11.81 | 194.7 ± 10.52 | 0.0130 | > 0.05 |
| P2 | 311.9 ± 13.50 | 137.8 ± 4.007 | 0.0031 | > 0.05 |
| P3 | 508.1 ± 6.954 | 257.7 ± 18.06 | 0.0002 | > 0.05 |
